# Splenic CD4^+^ T Cells in Progressive Visceral Leishmaniasis Show a Mixed Effector-Regulatory Phenotype and Impair Macrophage Effector Function through Inhibitory Receptor Expression

**DOI:** 10.1371/journal.pone.0169496

**Published:** 2017-01-19

**Authors:** Audrie A. Medina-Colorado, Elvia Y. Osorio, Omar A. Saldarriaga, Bruno L. Travi, Fanping Kong, Heidi Spratt, Lynn Soong, Peter C. Melby

**Affiliations:** 1 Department of Microbiology and Immunology, University of Texas Medical Branch, Galveston, Texas, United States of America; 2 Department of Internal Medicine, University of Texas Medical Branch, Galveston, Texas, United States of America; 3 Center for Tropical Diseases, University of Texas Medical Branch, Galveston, Texas, United States of America; 4 Institute for Human Infections and Immunity, University of Texas Medical Branch, Galveston, Texas, United States of America; 5 Department of Biochemistry and Molecular Biology, University of Texas Medical Branch, Galveston, Texas, United States of America; 6 Department of Preventive Medicine and Community Health, University of Texas Medical Branch, Galveston, Texas, United States of America; 7 Department of Pathology, University of Texas Medical Branch, Galveston, Texas, United States of America; INRS - Institut Armand Frappier, CANADA

## Abstract

Visceral leishmaniasis (VL), caused by infection with the intracellular protozoan *Leishmania donovani*, is a chronic progressive disease with a relentlessly increasing parasite burden in the spleen, liver and bone marrow. The disease is characterized by fever, splenomegaly, cachexia, and pancytopenia, and progresses to death if not treated. Control of *Leishmania* infection is mediated by Th1 (IFNγ-producing) CD4^+^ T cells, which activate macrophages to produce nitric oxide and kill intracellular parasites. However, despite expansion of CD4^+^ T cells and increased IFNγ expression in the spleen, humans with active VL do not control the infection. We used an experimental model of chronic progressive VL in hamsters, which mimics clinical and pathological features seen in humans, to better understand the mechanisms that lead to progressive disease. Transcriptional profiling of the spleen during chronic infection revealed expression of markers of both T cell activation and inhibition. CD4^+^ T cells isolated from the spleen during chronic progressive VL showed mixed expression of Th1 and Th2 cytokines and chemokines, and were marginally effective in controlling infection in an *ex vivo* T cell-macrophage co-culture system. Splenic CD4^+^ T cells and macrophages from hamsters with VL showed increased expression of inhibitory receptors and their ligands, respectively. Blockade of the inhibitory receptor PD-L2 led to a significant decrease in parasite burden, revealing a pathogenic role for the PD-1 pathway in chronic VL. PD-L2 blockade was associated with a dramatic reduction in expression of host arginase 1, but no change in IFNγ and inducible nitric oxide synthase. Thus, the expression of counter-regulatory molecules on splenic CD4^+^ T cells and macrophages promotes a more permissive macrophage phenotype and attenuates intracellular parasite control in chronic progressive VL. Host-directed adjunctive therapy targeting the PD-1 regulatory pathway may be efficacious for VL.

## Introduction

Visceral leishmaniasis (VL) is a neglected tropical disease caused by the protozoan parasite *Leishmania donovani* or *L*. *infantum* (= *L*. *chagasi*). Approximately 90% of infected individuals experience asymptomatic infection without overt evidence of disease. The remainder of infected individuals develop a chronically progressive infection that principally involves the spleen, liver and bone marrow. The outcome of infection is primarily determined by the host immune response, which is influenced by genetic makeup and environmental factors such as malnutrition [1, 2].

Active VL is the most serious form of leishmaniasis, accounting for approximately 500,000 cases annually [3, 4]. It is usually fatal if not treated, and even with treatment, the mortality rate can be as high as 20% [3, 5]. Most cases of VL are found in India, Bangladesh, Ethiopia, Sudan and Brazil. Clinical symptoms include chronic fever, loss of appetite, cachexia, and enlarged liver and spleen. Patients with progressive VL have pancytopenia, and loss of antigen-induced lymphoproliferative and Th1 cytokine responses in peripheral blood mononuclear cell cultures [6, 7].

In experimental models of VL, control of parasite replication requires an early and strong Th1 response with production of IL-12 and IFNγ [8, 9]. In human VL, there is a strong Th1 cytokine response (IFNγ, IL-1, IL-6, IL-12 and TNFα) at the site of infection [10, 11], but this is inexplicably unable to control the infection. Anti-inflammatory and type 2 cytokines (IL-4, IL-5, IL-10 and IL-13) are also increased in serum and spleen [11–13], and IL-10 in particular appears to have a dual effect of limiting inflammation and promoting permissiveness to parasite replication [14]. To further understand the pathogenesis of this disease, it is crucial to identify the cellular sources of protective and non-protective cytokines and to determine how T cells interact with infected macrophages to restrict or promote infection.

The golden Syrian hamster (*Mesocricetus auratus*) is an advantageous model to study the pathogenesis of VL because it mimics the chronic and progressive nature of human disease [15, 16]. Similar to humans, hamsters infected with *L*. *donovani* experience weight loss, hepatosplenomegaly, progressive parasite replication and ultimately death [17]. While it is clear that active VL is associated with a failure in cellular immunity to control parasite replication, the mechanisms behind this are unclear. As in humans, hamsters show increased splenic expression of the type 1 cytokines (IL-2, IL-12, IFNγ, TNFα) and the type 2 cytokines (IL-4, IL-10, IL-13, IL-21) [11, 17, 18].

The studies presented here focus on the nature and role of splenic CD4^+^ T cells in the hamster model of chronic, progressive VL. Transcriptional profiling of the infected spleen tissue identified a number of markers of T cell activation. A mixed cytokine response in spleen tissue was also evident in splenic CD4^+^ T cells. CD4^+^ T cells from chronically infected hamsters had the capacity to activate macrophages and induce parasite killing, but this was marginally effective relative to the killing induced by classical macrophage activation stimuli. Increased expression of T cell inhibitory markers, identified by transcriptional profiling of spleen tissues, led us to explore this as a potential contributor to suboptimal T cell effector function. We discovered that the splenic CD4^+^ T cell and macrophage populations expressed inhibitory receptors and ligands, respectively. Blocking PD-L2 led to a significant decrease in parasite burden in a splenic explant culture, revealing a pathogenic role for the PD-1 pathway in chronic VL.

## Materials and Methods

### Ethics statement

The animals used in this study were handled in strict accordance with the recommendations in the Guide for the Care and Use of Laboratory Animals of the National Institutes of Health. The protocol was approved by the Institutional Animal Care and Use Committee of the University of Texas Medical Branch, Galveston, Texas (protocol number 1101004). Animals were anesthetized during procedures with inhaled isoflurane and were euthanized by CO_2_ inhalation.

### Parasites

*Leishmania donovani* (MHOM/SD/001S-2D) promastigotes were cultured in M199 media supplemented with 0.1 mM adenine (in 50mM HEPES), 5 g/mL hemin (in 50% triethanolamine), 20% heat-inactivated fetal bovine serum (FBS), 100 U/mL penicillin, 100 mg/mL streptomycin at 26°C. Metacyclic promastigotes were isolated from early passage 7-day cultures by peanut agglutination as previously described [19]. Promastigote infectivity was maintained by regular *in vivo* passages through Syrian golden hamsters.

### Hamsters and *in vivo* infections

Outbred Syrian golden hamsters (*Mesocricetus auratus*) were obtained from Harlan Laboratories and were maintained and used according to the Guide for the Care and Use of Laboratory Animals of the National Institutes of Health. 6–8-week old hamsters were infected by intracardiac injection of 10^6^ metacyclic *L*. *donovani* promastigotes in 50 μL Dubelcco’s Modified Eagle’s Medium (DMEM) or Phosphate Buffered Saline (PBS). For co-culture experiments, an inbred Chester Beatty hamster colony was maintained in the animal resource center at the University of Texas Medical Branch. Inbred hamster litters were weaned at 3 weeks old and male or female hamsters used at 4–6 weeks of age. Experiments were set up using cells from sex-matched hamsters.

### Transcriptional profiling by RNA sequencing

Next generation sequencing of uninfected and 28-day infected spleen tissue (n = 5 hamsters per group) was performed. In short, total RNA was used to construct libraries for deep sequencing using the Illumina TruSeq RNA Sample Preparation Kit. Agilent Bioanalyzer confirmed the quality of the library and Truseq SBS kit v3 was used to sequence paired-end 50 base reads on an Illumina HiSeq 1000. Reads that aligned to the *L*. *donovani* BPK282A1 genome were removed and *de novo* assembly of a complete hamster transcriptome was performed with Trinity and BRANCH software using the Texas Advanced Computing Center (TACC) at the University of Texas at Austin. A false discovery rate (FDR) cutoff of <0.01 and fold change (FC) cutoffs of ≥2 or ≤-2 were used to identify differentially expressed transcripts. Gene identification was achieved by BLAST alignment to the *Rattus norvegicus* and *Mus musculus* reference genomes. The transcriptome data have been deposited in NCBI's Gene Expression Omnibus [20] and is accessible through GEO Series accession number GSE91187 (http://www.ncbi.nlm.nih.gov/geo/query/acc.cgi?acc=GSE91187). To explore the biological context of the differentially expressed genes, the upregulated (≥2 FC) or downregulated (≤-2 FC) transcripts (4360 total) were uploaded into WEB-based GEne SeT AnaLysis Toolkit (WebGestalt) online software using the *Mus musculus* reference genome and matched gene symbols [21]. WikiPathways Enrichment Analysis was run using default settings and a significance level cutoff of 0.01. Entrez Gene IDs were translated back to Gene symbols using UniProt resource database [22].

### Isolation of splenic CD4^+^ T cells

Spleens from uninfected and infected hamsters were collected in ice-cold RPMI 1640 medium supplemented with Glutamax (Gibco), 10% heat inactivated FBS, 0.5 mM EDTA (Gibco) and 0.6 μg DNase (Sigma). Spleens were digested for 10 minutes at 37°C by injecting with collagenase D (Roche) at 2 mg/mL in buffer containing (150 mM NaCl, 5 mM KCl, 1 mM MgCl_2_, 1.8 mM CaCl_2_, 10 mM Hepes pH 7.4). The tissue was further minced and strained through a 100μm cell strainer to obtain a single cell suspension that was plated in large tissue culture flasks for 30 minutes at 37°C in 5% CO_2_ in 10% FBS complete DMEM culture medium to remove adherent cells. The non-adherent cell population was collected and the procedure repeated. The cells were washed once in 10% FBS complete RPMI and resuspended in 1x red blood cell lysis buffer (0.2 mM NH_4_Cl, 0.01M NaHCO_3_, 0.1mM EDTA, pH 7.4) for 10 minutes and washed again with 10% FBS complete RPMI. Cells were labeled with anti-mouse CD4^+^ magnetic particles (clone GK1.5), resuspended in ice-cold separation buffer (1x PBS, 0.5% bovine serum albumin, 2mM EDTA, pH 7.2), and separated using a BD magnet following manufacturer’s protocol (BD iMag Cell Separation System, BD Biosciences). The enriched CD4^+^ cell population was resuspended in 10% FBS complete RPMI and counted for FACs staining and RNA isolation.

### Flow cytometry

Single cell suspensions of enriched CD4^+^ splenocytes obtained from 28-day infected and uninfected control hamsters were adjusted to a concentration of 5–10 × 10^5^ cells per 100μL of blocking buffer containing 2% normal mouse serum and 2% normal rat serum in PBS. Cells were stained with APC-Cy^™^7 conjugated rat anti-mouse CD4^+^ or isotype control (BD Biosciences) and FITC conjugated rat anti-human CD3 (clone CD3-12) or isotype control (AbD Serotec) for 30 minutes in the dark at 4°C followed by washing in PBS with 2% FBS and 0.1% sodium azide. For intracellular staining, cells were fixed/permeabilized using Foxp3/transcription factor staining buffer set and stained with PE-Cy5 conjugated anti-mouse/rat Foxp3 (clone FJK-16S) or isotype control, and eFluor 660 conjugated anti-human/mouse T-bet (clone 4B10) or isotype control (eBioscience). All flow cytometric analyses were performed on a Stratedigm SE520EX6 flow cytometer using software CellCapTure v3.1.0. Data were analyzed using FlowJo v10.0.7 (Treestar).

### Determination of gene expression by real-time RT-PCR

Spleen tissue or isolated CD4^+^ T cells from uninfected and 7-, 14-, 21- and/or 28-day infected hamsters were collected and RNA was isolated using the Qiagen RNeasy Mini Kit (for concentrations larger than 1 μg) or Ambion RNAqueous-Micro Total RNA Isolation Kit (for concentrations less than 1 μg RNA). Total RNA was DNase treated with Life Technologies Turbo DNA-Free kit and reverse transcribed into cDNA according to manufacturer’s protocol (High-Capacity cDNA Reverse Transcription Kit, Life Technologies). Primer sequences were designed using Genscript Primer Design Tool and the National Center for Biotechnology Information (NCBI) Primer-BLAST, which provided wider selection parameters. The Ensembl genome database was used to map exons and introns according to the mouse genome, and each primer set was designed to span an intron on the hamster target gene. Primer fidelity was confirmed by analysis of dissociation curves. The target genes for which primers were designed are detailed in S1 Table. Gene expression was determined in total spleen tissue, baby hamster kidney cells (BHK fibroblast cell line) and CD4^+^ splenocytes by SYBR green PCR on ViiA 7 Real-Time PCR System. Data was analyzed using comparative Ct method relative to uninfected BHK controls or uninfected hamster controls and using the 18S ribosomal RNA gene as the normalizer.

### Isolation of hamster bone marrow-derived macrophages

Femurs from uninfected hamsters were collected and bone marrow cells were flushed using GlutaMax RPMI 1640 culture medium (Gibco) supplemented with 10% heat inactivated FBS, 50 μM β-mercaptoethanol, 100 U/mL penicillin, 100 mg/mL streptomycin and 20 ng/mL recombinant human macrophage-colony stimulating factor (M-CSF) (eBioscience). Cells were adjusted to 8 x 10^6^/mL and cultured for 3 days at 37°C in 5% CO_2_ after which the culture medium was replenished and cells were allowed to differentiate for 3 more days and purity was determined by microscopy as previously described [23]. Cells were then washed with PBS and detached with Trypsin/EDTA (Gibco).

### IFNγ-induced macrophage activation and priming

Bone marrow-derived macrophages were plated and allowed to adhere overnight in 2% FBS complete RPMI medium. The next day, culture medium was replenished and cells were primed with IFNγ (10% v/v in supernatants from CHO cells expressing recombinant hamster IFNγ) [24] for 1–2 hours and then LPS (20 ng/μL) was added. For *in vitro* infections, stationary phase *L*. *donovani* promastigotes from 6–7 day old cultures were used at a parasite to cell ratio of 5:1 in 2% FBS complete RPMI medium. Parasites were allowed to be phagocytized for 4 hours and then the monolayer was carefully washed with pre-warmed medium to remove extracellular parasites. Media was replenished and cells were incubated for 48 hours at 37°C in 5% CO_2_.

### T cell—macrophage co-cultures

CD4^+^ T cells were isolated from control or chronically infected inbred hamster spleens as described. For co-cultures, CD4^+^ T cells from control or chronically infected hamsters were added to wells with uninfected or infected macrophages. For transwell assays, the macrophages were cultured in the bottom chamber, and purified CD4^+^ T cells were cultured in a maximum of 100 μL culture medium in top transwell inserts with 0.4 μm pore polycarbonate membrane (Corning). Co-cultures and transwell assays were set up with 5x10^5^ T cells and 1x10^5^ macrophages (ratio of 5:1). Cells were cultured for 1 and 48 hours at 37°C in 5% CO_2_. At each time point, culture medium was aspirated and cells were lysed for RNA isolation as described. For co-culture experiments, a *L*. *donovani* strain transfected with an episomal vector containing the Luciferase reporter gene was used [25]. To determine the parasite burden, the macrophages were lysed using Promega luciferase assay kit to determine parasite luciferase activity on the FluorStar Model 403 [18, 25]. The PD-1 pathway was blocked in *ex vivo* spleen cell cultures using mouse anti-PDL-2 antibody (B7-DC, R&D) and compared to the isotype control.

### Statistical analysis

Comparison between two groups was performed using Student’s *t* test (parametric) or Mann-Whitney test (non-parametric) depending on the normalcy of distribution. Comparison between more than 2 groups was performed using one-way ANOVA (parametric) or Kruskall-Wallis (non-parametric) with a correction for multiple comparisons. For experiments using 2 independent variables, comparisons were done using two-way ANOVA. Bonferroni’s multiple comparisons test was used. *p*-values <0.05 were considered significant. All analyses were conducted using GraphPad Prism version 5.04 for Windows or Mac (GraphPad Software, San Diego, California, USA).

## Results

### Progressive increase in parasite burden is associated with upregulation of genes in T cell activation pathways

**Fig 1:**
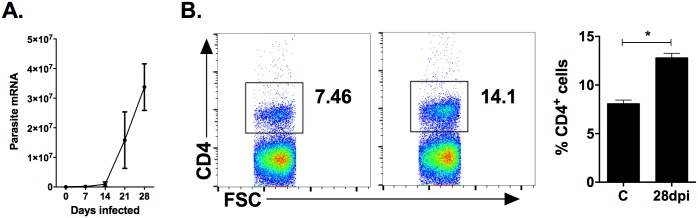
Chronic *L*. *donovani* infection leads to accumulation of CD4^+^ T cells in the spleen. (A) Splenic parasite burden was determined by real time RT-PCR of *L*. *donovani* 18s mRNA at 0, 7, 14, 21, and 28 days post-infection). (B) The frequency of CD4^+^ splenic T cells from uninfected and 28-day infected hamsters was determined by flow cytometry. Total lymphocytes were gated based on FSC and SSC. Shown is the frequency of CD4^+^ lymphocytes in total spleen cell population (n = 4–6 hamsters) *p<0.05.

We confirmed the dramatic increase in parasite burden over the course of the first 28 days in *L*. *donovani*-infected hamsters [16, 17] (Fig 1A). If allowed, the parasite burden would continue to increase until the animal’s death at 10–12 weeks post-infection [17]. To determine the effect on the splenic CD4^+^ T cell population, we compared uninfected with 28-day infected hamsters by flow cytometry. The percentage of CD4^+^ T cells in the spleen increased significantly during chronic stages of the disease (*p* = 0.036) (Fig 1B). To gain insight into the splenic gene expression involved in T cell function in progressive disease, we used unbiased transcriptional profiling (RNA sequencing) to identify pathways enriched in VL in 28 day infected hamsters. Input of both upregulated and down-regulated genes identified pathways related to T cell activation and effector function (S2 Table). From a set of manually curated genes known to be involved in CD4^+^ T cell activation or effector function we also found evidence of significant T cell activation at 28 days of infection (Table 1). Remarkably the upregulated genes showed a mix of CD4^+^ T cell activation/effector and exhaustion markers. The simultaneous increase in activation and exhaustion markers suggests dysfunctional T cell effector function and could explain the ineffective control of infection.

**Table 1:** Upregulated genes related to T cell activation and function ^a^.

Gene Symbol	Protein Name	FC ^c^
IFNG	Interferon gamma	52.2
LAG3 ^b^	Lymphocyte activation gene 3	27.3
PDCD1LG2 ^b^	Programmed cell death 1 ligand 2	26.9
CCR5	C-C chemokine receptor 5	19.8
TNFRSF4	OX40, Tumor necrosis factor receptor superfamily member 4	9.7
CTLA4 ^b^	Cytotoxic T-lymphocyte associated protein 4	7.5
TNF	Tumor necrosis factor alpha	6.8
IL12RB2	Interleukin 12 receptor subunit beta 2	4.8
CXCR3	C-X-C chemokine receptor 3	3.6
PDCD1 ^b^	Programmed cell death 1	3.3
TNFRSF9	CD137, Tumor necrosis factor receptor superfamily member 9	3.1
TBX21	Tbet, T-box transcription factor 21	3.0
PTPRC	CD45 antigen, protein tyrosine phosphatase receptor type c	2.7
TNFRSF14	CD270, Tumor necrosis factor receptor superfamily member 14	2.6
CD274 ^b^	Programmed cell death 1 ligand 1	2.6
CD44	CD44 antigen	2.4
IL2RB	Interleukin 2 receptor subunit beta	2.0
CD4	Cluster of differentiation 4	1.6

^**a**^ Manually curated list; False Discovery rate <0.01

^**b**^ Inhibitory receptors

^**c**^ Fold-change

### Mixed splenic cytokine profile in response to chronic VL

**Fig 2:**
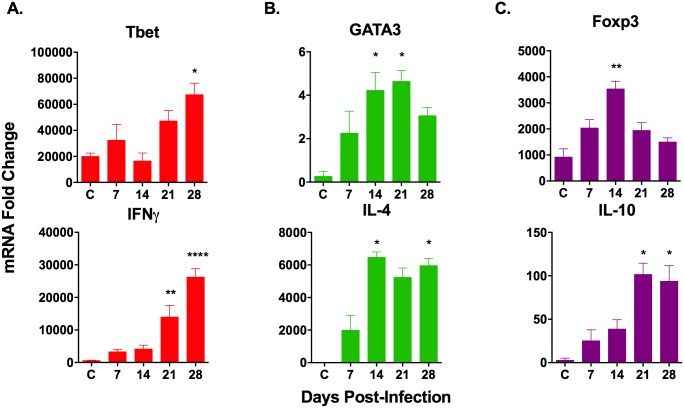
Splenic expression of markers of CD4^+^ T cell subpopulations over the course of chronic *L*. *donovani* infection. RNA was isolated from spleen tissue from uninfected controls (C) and hamsters infected for 7, 14, 21 and 28 days. Gene expression for (A) Th1 (B) Th2 and (C) Treg cells was determined by real time RT-PCR. Fold change was calculated relative to basal gene expression in uninfected baby hamster kidney (BHK) cell line. Figures are representative of at least 3 independent experiments with 3–6 animals per experiment. *p<0.05, **p<0.01, ****p<0.0001. Th1-associated genes indicated by the color red, Th2-associated genes indicated by the color green, Treg-associated genes indicated by the color purple.

To further characterize the functional capacity of splenic T cells, we first determined mRNA expression of Th1, Th2, and regulatory CD4^+^ T cell (Treg) markers in the spleens of *L*. *donovani*-infected hamsters over the course of early VL. We found mRNA expression of the Th1 transcription factor, T-bet, and the Th1 cytokine, IFNγ, to increase over the course of infection (Fig 2A). However, Th1 markers expression was preceded (at 14 days post-infection) by expression of the master regulator transcription factor for Th2 cells, GATA3, and the associated Th2 cytokine, IL-4 (Fig 2B). In addition to the upregulation of Th1 and Th2 markers, mRNA of the transcription factor that regulates the differentiation of Tregs, Foxp3, was transiently increased at 14 days post-infection (Fig 2C). This suggests a transient regulatory CD4^+^ T cell response early in infection before Th1 CD4^+^ T cells significantly increase. The regulatory cytokine produced by Treg, Th2 and some Th1 cells, IL-10, showed significant increase in the spleen at 21 and 28 days post-infection (Fig 2C). Although cells other than CD4^+^ T cells can produce these cytokines, these data prompted us to examine their expression in purified CD4^+^ T cells during the course of infection.

### Splenic CD4^+^ T cells display a mixed Th1 and Th2 profile during chronic VL

**Fig 3:**
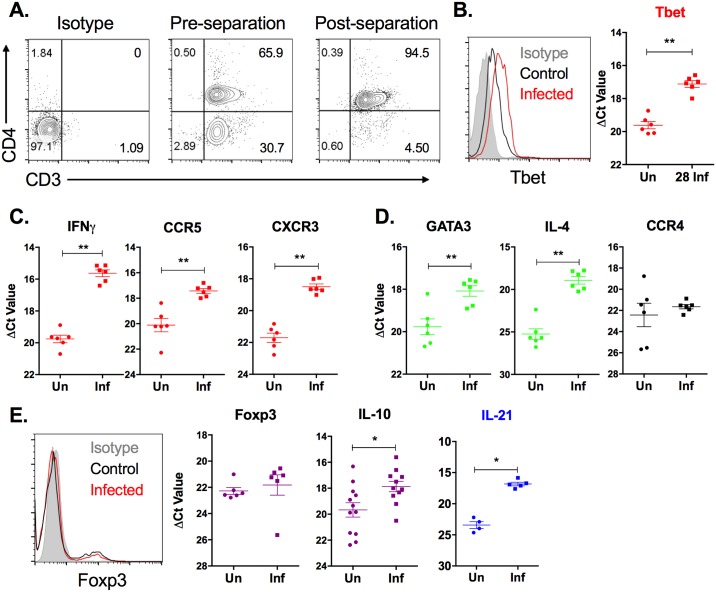
Splenic CD4^+^ T cell expression of markers of T cell subpopulations over the course of chronic *L*. *donovani* infection. CD4^+^ T cells were isolated from spleen tissue from uninfected (Un) or 28-day infected (Inf) hamsters by positive selection. (A) The post-separation purity of CD3^+^CD4^+^ T cells was >90% in multiple independent experiments. (B-E) RNA was isolated from the purified splenic CD4^+^ T cell population and mRNA expression of markers of Th1 (B, C), Th2 (D), and Treg cells (E) was determined by real time RT-PCR. Results are expressed as a relative fold-increase between experimental samples and uninfected BHK cells. Shown is the mean and SEM of a single experiment representative of 2 independent experiments from 6 hamsters per group. Expression of (B) T-bet and (E) Foxp3 was verified in CD4^+^ splenocytes by flow cytometry. Data is representative of at least 2 independent experiments. *p<0.05, **p<0.01. Th1-associated genes indicated by the color red, Th2-associated genes indicated by the color green, Treg-associated genes indicated by the color purple, Th2/Treg-associated genes indicated by the color black, Th1/Th2-associated genes indicated by the color blue.

We determined the phenotype of CD4^+^ T cells that accumulate in the spleen during chronic VL by investigating mRNA expression in isolated splenic CD4^+^ T cells. CD4^+^ splenocytes were enriched using magnetic particles conjugated with anti-CD4 antibody. This yielded a >90% pure CD3^+^CD4^+^ population that was used for real time RT-PCR analysis (Fig 3A). In this purified CD4^+^ T cell population we found a significant increase in mRNA and protein expression of the transcription factor, T-bet (Fig 3B). The mRNAs for the type 1 cytokine IFNγ and chemokine receptors associated with Th1 cells, CCR5 and CXCR3, were also significantly increased (Fig 3C). The Th2 transcription factor, GATA3, and Th2 cytokine, IL-4, were also significantly increased in splenic CD4^+^ T cells in VL, but the chemokine receptor commonly expressed on Th2 and Treg cells, CCR4, was not increased (Fig 3D). Consistent with what was found in spleen tissue at 28 days post-infection (Fig 2C), the transcription factor Foxp3, showed no increase in mRNA or protein in splenic CD4^+^ T cells at this time point (Fig 3E). IL-10 and IL-21, which can be produced by multiple T cell subsets and are thought to have a disease-promoting effect in active VL [26], were significantly upregulated in splenic CD4^+^ T cells in infected animals (Fig 3E). Collectively, our data indicate that splenic CD4^+^ T cells exhibit markers of both Th1 and Th2 development during chronic VL. Whether this Th1/Th2 profile is indicative of a double positive T cell phenotype or there are two separate T cell populations accumulating simultaneously could not be determined because antibodies against these markers in hamsters are not available.

### Expression of chemokine ligands and receptors is increased in spleen tissue, splenic macrophages and CD4^+^ T cells in VL

**Fig 4:**
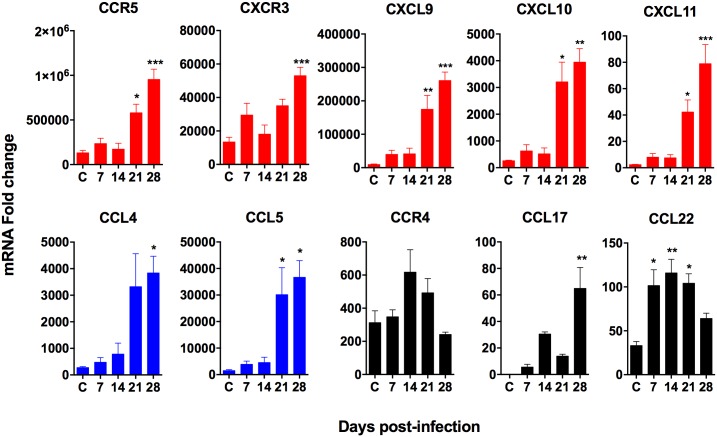
Chemokine receptor and ligand mRNA expression in hamster spleen tissue over the course of chronic *L*. *donovani* infection. mRNA expression of type 1, type 2 and regulatory type chemokine ligands and their receptors was determined by real time RT-PCR in spleen tissue from uninfected controls (C) or hamsters infected for 7, 14, 21 and 28 days. Results are expressed as a relative fold-increase between experimental samples and uninfected BHK cells. Shown is the mean and SEM of a single experiment representative of 2 independent experiments from 3–6 hamsters per time point. *p<0.05; **p<0.01, ***p<0.001. Th1-associated genes indicated by the color red, Th2/Treg-associated genes indicated by the color black, Th1/Th2-associated genes indicated by the color blue.

Because of the accumulation of CD4^+^ T cells in the spleen during VL, we investigated the expression of chemokine ligands and their receptors known to selectively recruit Th1, Th2, or Treg CD4^+^ cells in the spleens of hamsters with VL. We found mRNAs of the type 1 chemokine ligands and their receptors (CCL4, CCL5, CCR5, CXCL9, CXCL10, CXCL11 and CXCR3) were increased at 21–28 days post-infection compared to uninfected controls (Fig 4). Expression of the type 2 chemokine ligands, CCL17 and CCL22 increased as early as 7 days post-infection and remained upregulated up to 21 days of infection (Fig 4), but somewhat surprisingly their receptor, CCR4, was not (Fig 4). We did not measure expression of CCR8, which is a secondary receptor for CCL17 and CCL22.

**Fig 5:**
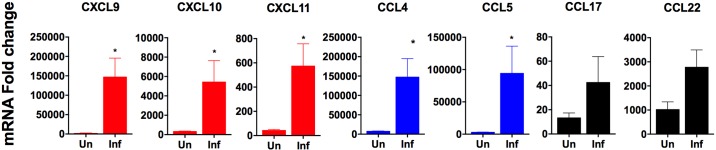
Chemokine ligand mRNA expression in splenic macrophages from chronically infected hamsters. Splenic macrophages were isolated from uninfected (Un) or 28-day infected (Inf) hamsters. mRNA expression of type 1, type 2 and regulatory type chemokine ligands was determined by real time RT-PCR. Results are expressed as a relative fold-increase compared to uninfected BHK cells. Shown is the mean and SEM of a single experiment representative of 2 independent experiments from 4 hamsters per time point. Student’s t-test was performed to compare control uninfected (C) and chronically 28 day infected (28dpi) samples. *p<0.05. Th1-associated chemokines indicated by the color red, Th1/Th2-associated chemokines indicated by the color blue, Th2/Treg-associated chemokines indicated by the color gray.

In order to determine the cellular source of the chemokine ligands, we next examined their expression in splenic macrophages isolated from chronically infected hamster spleens. The chemokine ligands that bind the Th1-associated chemokine receptors CXCR3 (CXCL9, CXCL10, CXCL11) and CCR5 (CCL4 and CCL5) were significantly upregulated in splenic macrophages during VL (Fig 5). The Th2-attracting chemokines, CCL17 and CCL22, showed a non-significant trend of increased expression. The increase in mixed chemokine ligands in this isolated cell population is consistent with the data from total spleen tissues (Fig 4). Together, these data suggests splenic macrophages are a significant source of T cell-attracting chemokines and may account for the accumulation of type 1 and type 2 CD4^+^ T cell subsets in the spleen during chronic VL.

### Splenic CD4^+^ T cells from chronically infected animals modestly reduce intracellular parasite burden *in vitro*

**Fig 6:**
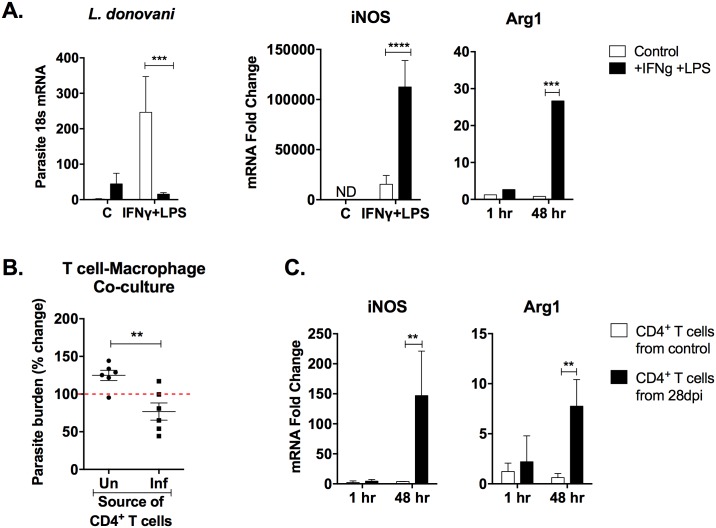
Control of intracellular parasite burden by IFNγ-LPS and splenic CD4^+^ T cell-mediated macrophage activation. (A) Hamster bone marrow-derived macrophages were primed with IFNγ before being triggered with LPS and infected with *L*. *donovani*. Parasite burden and macrophage activation were determined by measuring mRNA expression (real time RT-PCR) of *Leishmania* 18s, iNOS and Arg1, respectively. (B) Bone marrow-derived macrophages from uninfected hamsters were infected *in vitro* with luciferase-transfected *L*. *donovani* promastigotes and co-cultured with CD4^+^ T cells purified from uninfected or 28-day infected hamsters for 48 hours. The intracellular parasite burden in CD4^+^ T cell-macrophage co-cultures was determined using relative luminescent unit values and is presented as the percent increase or decrease from the baseline (1 hr) parasite burden (100%) for each group. (C) iNOS and Arg1 mRNA expression in CD4^+^ T cell-macrophage co-cultures was determined by real time RT-PCR at baseline (1 hr) and 48 hours later. Results are expressed as a relative fold change in comparison to the initial time point of each treatment group. All data shown is representative of at least 2 independent experiments with 6 replicates per group. ND = Not Detected. **p<0.01, ***p<0.001, ****p<0.0001.

Macrophages stimulated with IFNγ have a classically activated (M1) phenotype, characterized by production of reactive oxygen and nitrogen species [27–29]. Nitric oxide (NO) generated by inducible nitric oxide synthase (iNOS) is the primary effector of intracellular parasite killing. The relentlessly progressive disease in humans and hamsters in the face of Th1 cell accumulation and IFNγ production suggests that macrophages are not being effectively activated to kill the parasite [30, 31]. To investigate this, we first tested the effector function of hamster macrophages in an *in vitro* activation system. Bone marrow-derived macrophages were first primed with IFNγ for an hour before LPS stimulation and *L*. *donovani* infection. The primed-activated macrophages showed a dramatic increase in the uptake of parasites compared to unstimulated control macrophages after 1 hour (Fig 6A). After 48 hours, the IFNγ- and LPS-stimulated macrophages showed a significant (12-fold) reduction in parasite burden (Fig 6A) that was accompanied by increased expression of iNOS and Arg1 (Fig 6A). In contrast, there was no decrease in parasite load in the unactivated macrophages. These data suggest that in a controlled *in vitro* system, hamster macrophages are fully capable of upregulation of iNOS and intracellular parasite killing. We next evaluated the capacity of splenic CD4^+^ T cells, isolated from uninfected controls and hamsters with VL, to activate *in vitro* infected macrophages to kill intracellular parasites in a co-culture assay. To avoid any possible detection of parasite DNA carried with the CD4^+^ T cells isolated from infected hamsters, we used a luciferase-transfected *L*. *donovani* strain for the *in vitro* macrophage infections and measured parasite burden by luciferase activity. After 48 hours, the parasite burden was modestly (1.5-fold) but significantly decreased in the infected macrophages co-cultured with the CD4^+^ T cells isolated from hamsters with VL compared to those from uninfected hamsters (Fig 6B). This was accompanied by a significant increase in iNOS expression (Fig 6C), although its upregulation was substantially less than that observed in IFNγ/LPS-stimulated macrophages (Fig 6A). Notably, there was also an increase in Arg1 expression, a marker of alternative (M2) macrophage activation, when the infected macrophages were co-cultured with CD4^+^ T cells from hamsters with VL (Fig 6C). Collectively, these data indicate that the mixed phenotype of splenic CD4^+^ T cells in VL leads to expression of markers of both M1 (iNOS) and M2 (Arg1) activation that marginally reduces intracellular parasite burden.

### Increased splenic expression of T cell exhaustion markers in VL

**Fig 7:**
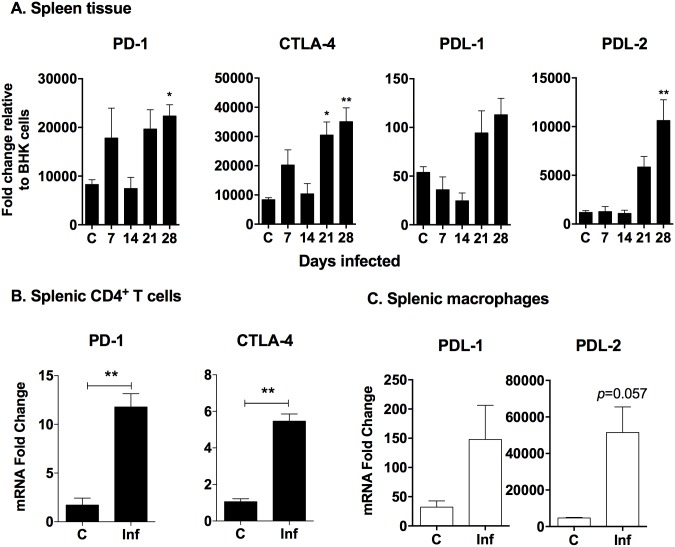
Increased splenic expression of inhibitory markers in chronic VL. mRNA expression for inhibitory markers was determined by real time RT-PCR for hamster (A) total spleen tissue (B) splenic CD4^+^ T cells and (C) splenic macrophages. Results are expressed as relative fold-increase over uninfected BHK cells. Shown is the mean and SEM of a single experiment representative of 2 independent experiments from 3–6 hamsters per time point. *p<0.05; **p<0.01.

Cellular immune function is ineffective in abating the relentless disease progression in VL, and CD4^+^ T cells co-cultured with *in vitro* infected macrophages uncovered only modest T cell effector activity (Fig 6C). Our transcriptional profiling data in spleen tissue identified the upregulation of a number of receptors/ligands that function to inhibit T cell responses and are associated with T cell exhaustion [32] (Table 1). We therefore investigated markers of T cell exhaustion in splenic CD4^+^ T cells during chronic VL. We found that the inhibitory receptors PD-1 and CTLA-4, and the ligands for PD-1 (PD-L1 and PD-L2), increased in spleen tissues as the disease progressed (Fig 7A). PD-1 and CTLA-4 were also significantly increased in isolated splenic CD4^+^ T cells from 28-day infected animals (Fig 7B). Splenic macrophages isolated from 28-day infected hamsters showed an insignificant increase in PD-L1 (*p* = 0.3429), and a trend toward increased PD-L2 expression (*p* = 0.0571) (Fig 7C). Collectively, these data suggest that inhibitory signaling between infected macrophages and CD4^+^ T cells may occur in the spleen during chronic VL.

### Blockade of the PD-1 pathway leads to enhanced parasite control

**Fig 8:**
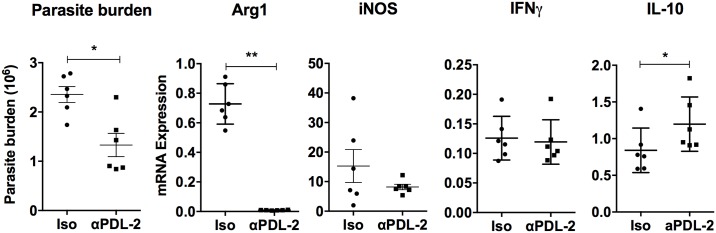
*Ex vivo* blockade of PD-L2 reduces parasite burden. Spleen cells from 28-day chronically infected hamsters were cultured with αPD-L2 antibody or isotype control for 48 hours. The parasite burden (18s mRNA expression) and expression of iNOS, IFNγ, Arg1 and IL-10 were determined by real time RT-PCR. Results are expressed as a relative fold-increase of the αPD-L2 antibody or isotype control groups compared to infected splenocytes at 0 hours. Shown is the mean and SEM of two independent experiments with 6 replicates per group.

To investigate the functional significance of the increased inhibitory molecules on macrophages and CD4^+^ T cells in the model of progressive VL, we blocked the PD-1 pathway with anti-PD-L2 antibody in *ex vivo* spleen cell cultures (containing both macrophages and T cells) from hamsters with VL. PD-L2 was targeted because of its increased expression relative to PD-L1 in the spleen and splenic macrophages. We found the parasite burden was significantly reduced after 48 hours incubation with anti-PD-L2 antibody compared to the isotype control (Fig 8). This was associated with a striking decrease in Arg1 mRNA expression, but no change in iNOS or IFNγ expression and a slight increase in IL-10. This suggests that blockade of the PD-1/PD-L2 interaction diminishes T cell-induced, disease-promoting arginase expression by macrophages and that the reduced parasite burden is not mediated by reduced IL-10. Together, these findings suggest activation of the PD-1 signaling pathway in the spleen plays a pathological role in progressive VL in the hamster model, and that this can be reversed by blockade of this inhibitory pathway.

## Discussion

Individuals with subclinical *L*. *donovani* infection demonstrate robust CD4^+^ T cell responses with IFNγ-mediated macrophage activation and parasite control [33, 34]. However, during active VL cultures of purified peripheral blood mononuclear cells show T cell unresponsiveness to *Leishmania* antigens [6, 7]. These studies may not be fully representative of T cell function because parasite-responsive cells are more likely to be found at the sites of visceral infection (e.g. spleen). Using an experimental model that closely mimics active human disease, we found an increase in accumulation of CD4^+^ T cells with a mixed Th1/Th2 cytokine/chemokine profile in the spleen during chronic VL. This corroborates studies of cytokine expression in human VL and in other models of non-healing leishmaniasis [11, 35]. The expression of T-bet and IFNγ by splenic CD4^+^ T cells in our VL model adds to the growing evidence of a strong type-1 (IFNγ) splenic immune response during active VL. But the question remains: why are macrophages in this Th1 environment unable to control *L*. *donovani* infection? Splenic CD4^+^ T cells isolated from hamsters with VL (28 days post-infection) showed marginal capacity to inhibit intracellular parasite growth in co-cultured *in vitro* infected macrophages. This suggests that co-expression of regulatory cytokines (IL-4, IL-10 and IL-21) and/or expression of inhibitory receptors in the CD4^+^ T cell population contributes to suboptimal macrophage activating capacity of the splenic CD4^+^ T cells.

Splenic CD4^+^ T cell expression of IL-4, IL-10 and IL-21 was significantly increased in hamsters with VL. IL-4 has a major role in the susceptibility of mice to experimental *L*. *major* infection (reviewed in [36]), either through inhibiting macrophage generation of effector molecules [37] and/or polarization of macrophages to produce arginase 1 [18, 38]. However, in mice that have a non-progressive *L*. *donovani* infection, IL-4 does not contribute to host susceptibility [39, 40]. While IL-4 is increased in the plasma or serum [11, 12, 41] and spleens [11] of patients with active VL, its role in pathogenesis of human VL has not been defined. Our previous finding that pathologic parasite-induced arginase is amplified by IL-4 in experimental VL suggests that IL-4-producing CD4^+^ T cells may contribute to impaired control of infection. IL-10 has been more clearly demonstrated to have a role in VL pathogenesis [14]. Neutralization of IL-10 in spleen cell explant cultures from subjects with VL led to reduced parasite load [42]. The identity of cells that produce IL-10 in human VL is controversial. IL-10 producing CD4^+^ T cells has been characterized in the VL mouse model as Foxp3^−^ cells [43, 44]. In humans, Nylen, et al, found the source of splenic IL-10 to be a CD4^+^Foxp3^−^ cell population [11]. Contrary to this, others found CD25^+^Foxp3^+^ Treg cells accumulated in the spleen in response to *Leishmania* antigen and produced IL-10 [45, 46]. We did not find sustained expansion of a CD4^+^Foxp3^+^ regulatory cell population, despite prominent expression of the regulatory cytokine IL-10. CD4^+^ T cells that produce both IFNγ and IL-10 have been described in human VL [11] and in chronic infection with *Toxoplasma gondii* and *Mycobacterium tuberculosis* [47, 48]. Distinction of individual cytokine-producing CD4^+^ T cells is not possible in the hamster model because of the lack of antibody reagents for multicolor flow cytometry. Splenic CD4^+^ T cells also expressed increased IL-21 in our model. Co-expression of splenic IL-10 and IL-21, and IL-21-mediated induction of IL-10, was shown in patients with VL [26]. Additionally, IL-21 could promote infection via M2 polarization of macrophages [49].

We found increased mRNA expression of PD-1, CTLA-4, PD-L1 and PD-L2 in chronically infected hamster spleen tissues, and increased expression of the inhibitory receptors CTLA-4 and PD-1 in the splenic CD4^+^ T cell population. Blockade of PD-L2 in an *ex vivo* spleen cell explant culture from hamsters with VL effected a reduction in parasite burden. This suggests a pathogenic role for the PD-1 pathway in this model of progressive VL. PD-1 and CTLA-4 are markers of T cell exhaustion, which multiple pathogens utilize to their benefit to establish chronic infection (reviewed in [32]). Their expression has been shown to increase soon after T cell activation is initiated. With chronic stimulation of T cells, the regulatory functions of CTLA-4 and PD-1 control overly aggressive T cell responses that could be damaging to the host. These inhibitory receptors lead to the gradual loss of activation and expansion of T cells, decreased cytokine production, and at the extreme, clonal deletion of the population [32]. T cell exhaustion has typically been demonstrated for CD8^+^ T cells as a result of long-term antigen stimulation in chronic viral infections [50], but CD4^+^ T cells and B cells [51] can also express PD-1 and CTLA-4. An increase in exhaustion markers was demonstrated in several models of VL [52–54]. CD8^+^ T cell exhaustion markers were described in patients with active VL, however, blockade of CTLA-4 and PD-1 did not recover CD8^+^ T cell responses to soluble *Leishmania* antigen or increase parasite killing in *ex vivo* cultures of spleen cells [53]. Mice infected with *L*. *donovani* showed evidence of progressive CD8^+^ T cell dysfunction, and *in vivo* blockade of PD-L1 improved CD8^+^ T cell survival and reduced the splenic parasite burden [54]. Increased expression of exhaustion markers in CD4^+^ T cells from dogs with chronic VL was found, and blockade of PD-L1 rescued *in vitro* CD4^+^ T cell proliferation and IFNγ production [52].

Most studies have focused on the effects of inhibitory receptors on T cell effector function, however, few studies have investigated their effects on the antigen presenting cell or phagocyte. Recently it was shown that engagement of the inhibitory receptors on macrophages and dendritic cells delivers a suppressive signal from the T cell to the myeloid cell [55–57]. Thus, the interaction between PD-1 and PD-L1/2 is likely to have a bi-directional effect. In support of this, we provide the evidence that blockade of PD-1/PD-L2 in spleen cell cultures from hamsters with VL led to decreased expression of arginase 1, which we demonstrated previously to promote macrophage susceptibility and VL progression [18, 23]. This is yet another subversive mechanism that renders macrophages unresponsive to antimicrobial activation [11, 58–60].

In summary, we describe for the first time the splenic CD4^+^ T cell phenotype in an experimental model of chronic progressive VL. We demonstrate that CD4^+^ T cells in hamster VL have a mixed cytokine/chemokine profile and marginal capacity to induce parasite killing in *in vitro* infected macrophages. Splenic CD4^+^ T cells also expressed regulatory cytokines, such as IL-10 and IL-21, and inhibitory receptors typically associated with T cell exhaustion. Remarkably, blockade of the PD-1/PD-L2 pathway in *ex vivo* spleen cell explant cultures inhibited host arginase expression and enhanced parasite control. Whether the protective effect mediated by PD-L2 blockade is through a direct action on macrophages or an indirect action through T cell signals is unknown. The fact that there was no difference in IFNγ expression suggests that the killing effect is not mediated by an increase in this Th1 cytokine, but through other mechanisms. Further investigation into the role of inhibitory receptors/ligands on the interaction between CD4^+^ T cells and macrophages is warranted.

## Supporting Information

S1 Table Hamster primer sequences designed for real time RT-PCR. (DOCX)

S2 Table Pathway analysis. (DOCX)
