# Supplementary material for: Splenic CD4+ T Cells in Progressive Visceral Leishmaniasis Show a Mixed Effector-Regulatory Phenotype and Impair Macrophage Effector Function through Inhibitory Receptor Expression
Source: PLoS One. 2017 Jan 19;12(1):e0169496. doi: 10.1371/journal.pone.0169496 (PMC5245871; doi:10.1371/journal.pone.0169496)
Supplement: S1 Table — (DOCX) [file pone.0169496.s001.docx]

TABLE S1. Hamster primer sequences designed for real time RT-PCR

| Target gene | Primer sequences |
| --- | --- |
| Tbet | *For:* 5’ – ACA AGG GGG CTT CCA ACA AT – 3’  *Rev:* 5’ – CAG CTG AGT GAT CTC GGC AT – 3’ |
| GATA3 | *For:* 5’ – GAA GGC AGG GAG TGT GTG AA – 3’  *Rev:* 5’ – GTC TGA CAG TTC GCA CAG GA – 3’ |
| Foxp3 | *For:* 5’ – AGG TCT TCG AGG AGC CAG AA – 3’  *Rev:* 5’ – GCC TTG CCC TTC TCA TCC A – 3’ |
| CCR5 | *For:* 5’ – TGT GAC ATC CGT TCC CCC T – 3’  *Rev:* 5’ – GGC AGG GTG CTG ACA TAC TA – 3’ |
| CXCR3 | *For:* 5’ – CAA GTG CCA AAG CAG AGA AGC – 3’  *Rev:* 5’ – CAA AGT CCG AGG CAT CTA GCA – 3’ |
| CCR4 | *For:* 5’ – GCT TGG TCA CGT GGT CAG TG – 3’  *Rev:* 5’ – GTG GTT GCG CTC CGT GTA G – 3’ |
| CXCL9 | *For*: 5’ – TGG GTA TCA TCC TCC TGG AC – 3’  *Rev*: 5’ – AAT GAG GAC CTG GAG CAA AC – 3’ |
| CXCL10 | *For:* 5’ – TGG AAA TTA TTC CTG CAA GTC A – 3’  *Rev*: 5’ – GTG ATC GGC TTC TCT CTG GT – 3’ |
| CXCL11 | *For:* 5’ – TGG CTG TGA TCA GTT GTG CT – 3’  *Rev:* 5’ – GTG CTT TCA GGG TAA CAA TCA CT – 3’ |
| CCL4 | *For:* 5’ – TCT CTC TCC TCC TGT TCG TGG – 3’  *Rev:* 5’ – TTT GCT TGC CTT TTC TGG TCA – 3’ |
| CCL5 | *For:* 5’ – CTA CGC TCC TTC ATC TGC CTC – 3’  *Rev:* 5’ – CCT TCG GGT GAC AAA AAC GAC – 3’ |
| CCL17 | *For:* 5’ – GTG CTG CCT GGA GAT CTT CA – 3’  *Rev:* 5’ – TGG CAT CCC TGG GAC ACT – 3’ |
| CCL22 | *For:* 5’ – CGT GGC TCT CAT CCT TCT TGC – 3’  *Rev:* 5’ – CAG ATG CTG TCT TCC ACG TTG G – 3’ |
| IL-4 | *For:* 5’ – CCT GCT CTG CCT TCT AGC AT – 3’  *Rev:* 5’ – GCC CTG CAG ATG AGG TCT TT – 3’ |
| IFNγ | *For*: 5’ – AAT ATC TTG ACG AAC TGG CAA A – 3’  *Rev*: 5’ – CCT TCA AGG CTT CAA AGA GTT T – 3’ |
| IL-10 | *For:* 5’ – TAA GGG TTA CTT GGG TTG CC – 3’  *Rev:* 5’ – TTC ACC TGT TCC ACA GCC TTG – 3’ |
| PD-1 | *For:* 5’ – CTG AAA AGG GTT AAG CCA GC – 3’  *Rev:* 5’ – GCC TCC AGG ATT CTC TCT GTT – 3’ |
| PD-L1 | *For:* 5’ – TGA TCA TCC CAG ACC CGC TC – 3’  *Rev:* 5’ – CTC CTC GAA CTG CGT ATC GT – 3’ |
| PD-L2 | *For:* 5’ – CAG TAC CGC TGT CTG GTC AT – 3’  *Rev:* 5’ – CTT CAG GGG TCC TGA TGT GG – 3’ |
| CTLA-4 | *For:* 5’ – GTT ATT GAA CCA GAA CCA TGC CC – 3’  *Rev:* 5’ – ACC CCT GTT GTA AGG GGA CT – 3’ |
