# Supplementary material for: Splenic CD4+ T Cells in Progressive Visceral Leishmaniasis Show a Mixed Effector-Regulatory Phenotype and Impair Macrophage Effector Function through Inhibitory Receptor Expression
Source: PLoS One. 2017 Jan 19;12(1):e0169496. doi: 10.1371/journal.pone.0169496 (PMC5245871; doi:10.1371/journal.pone.0169496)
Supplement: S2 Table — (DOCX) [file pone.0169496.s002.docx]

TABLE S2. Pathway analysis of upregulated T cell activation and function genes^a^

| **Pathway** | **Gene Symbol** | **Name** | **FC^b^** |
| --- | --- | --- | --- |
| **Type II interferon signaling (IFNγ)** | Ifng | Interferon gamma | 52.2 |
| *Adjusted p value = 2.5E-9* | Cxcl9 | C-X-C motif chemokine 9 | 29.4 |
|  | Cxcl10 | C-X-C motif chemokine 10 | 12.9 |
|  | Gbp2b | Guanylate binding protein 1 | 11.2 |
|  | Irf2 | Interferon regulatory factor 2 | 6.6 |
|  | Socs1 | Suppressor of cytokine signaling 1 | 5.3 |
|  | Socs3 | Suppressor of cytokine signaling 3 | 4.4 |
|  | Cybb | Cytochrome b-245 heavy chain | 4.1 |
|  | Isg15 | Ubiquitin like protein ISG15 | 3.8 |
|  | Stat1 | Signal transducer and activator of transcription 1 | 3.4 |
|  | Irf1 | Interferon regulatory factor 1 | 3.2 |
|  | Ifit2 | Interferon induced protein with tetracopeptide repeats 2 | 2.8 |
|  | Tap1 | Antigen peptide transporter 1 | 2.7 |
|  | Icam1 | Intercellular adhesion molecule 1 | 2.7 |
|  | Stat2 | Signal transducer and activator of transcription 2 | 2.1 |
|  | Irf4 | Interferon regulatory factor 4 | 2 |
|  |  |  |  |
| **T cell receptor signaling** | Tuba4a | Tubulin alpha-4A chain | 601.1 |
| *Adjusted p value = 7.67E-7* | Fyn | Proto-oncogene c-Fyn | 10.1 |
|  | Src | Neuronal proto-oncogene tyrosine-protein kinase Src | 10.1 |
|  | Sos2 | Son of sevenless homolog 2 | 7.2 |
|  | Sh2d2a | SH2 domain containing protein 2A | 6.5 |
|  | Skap1 | Src kinase-associated phosphoprotein 1 | 4 |
|  | Abi1 | Abelson interactor 1 | 4 |
|  | Cd8a | T cell surface glycoprotein CD8 alpha | 3.8 |
|  | Cebpb | CCAAT/enhancer-binding protein beta | 3.7 |
|  | Stat1 | Signal transducer and activator of transcription 1 | 3.4 |
|  | Ptk2b | Protein-tyrosine kinase 2-beta | 3 |
|  | Tubb5 | Tubulin beta-5 chain | 2.8 |
|  | Cd2 | T cell surface antigen CD2 | 2.7 |
|  | Ptprc | Protein tyrosine phosphatase receptor type C | 2.7 |
|  | Zap70 | Tyrosine-protein kinase zeta chain associated protein-70 | 2.6 |
|  | Stat5a | Signal transducer and activator of transcription 5A | 2.2 |
|  | Stat5b | Signal transducer and activator of transcription 5B | 2.2 |
|  | Lck | Proto-oncogene tyrosine-protein kinase LCK | 2.2 |
|  |  |  |  |
| **Pathway** | **Gene Symbol** | **Name** | **FC^b^** |
| **IL-2 signaling** | Plcb1 | 1-phosphatidylinositol 4,5-bisphosphate phosphodiesterase beta-1 | 347.1 |
| *Adjusted p value = 9.6E-6* | Fyn | Tyrosine protein kinase Fyn | 10.1 |
|  | Socs1 | Suppressor of cytokine signaling 1 | 5.3 |
|  | Socs3 | Suppressor of cytokine signaling 3 | 4.4 |
|  | Ets2 | E26 avian leukemia oncogene 2,3' domain | 3.7 |
|  | Stat1 | Signal transducer and activator of transcription 1 | 3.4 |
|  | Ptk2b | Protein tyrosine kinase 2 beta | 3 |
|  | Icam1 | Intercellular adhesion molecule 1 | 2.7 |
|  | Stat5a | Signal transducer and activator of transcription 5 alpha | 2.2 |
|  | Stat5b | Signal transducer and activator of transcription 5 beta | 2.2 |
|  | Lck | Proto-oncogene tyrosine-protein kinase LCK | 2.2 |
|  | Hsp90aa1 | Heat shock protein 90 alpha class A member 1 | 2.1 |
|  | Il2rb | Interleukin 2 receptor subunit beta | 2 |
|  |  |  |  |
| **IL-4 signaling** | Fyn | Tyrosine protein kinase Fyn | 10.1 |
| *Adjusted p value = 2.0E-4* | Src | Neuronal proto-oncogene tyrosine-protein kinase Src | 10.1 |
|  | Socs1 | Suppressor of cytokine signaling 1 | 5.3 |
|  | Ncf1 | Neutrophil cytosol factor 1 | 4.4 |
|  | Socs3 | Suppressor of cytokine signaling 3 | 4.4 |
|  | Stat1 | Signal transducer and activator of transcription 1 | 3.4 |
|  | Il13ra1 | Interleukin 13 receptor subunit alpha 1 | 3.2 |
|  | Bcl2l1 | B cell lymphoma 2 like protein | 3.1 |
|  | Stat5a | Signal transducer and activator of transcription 5 alpha | 2.2 |
|  | Lck | Proto-oncogene tyrosine-protein kinase LCK | 2.2 |

**^a^** False Discovery rate <0.01

**^b^** Fold-change
